# Supplementary material for: Integrating a host biomarker with a large language model for diagnosis of lower respiratory tract infection
Source: Nat Commun. 2025 Dec 16;16:10882. doi: 10.1038/s41467-025-66218-5 (PMC12708615; doi:10.1038/s41467-025-66218-5)
Supplement: Supplementary file 1 — Supplementary Information [file 41467_2025_66218_MOESM1_ESM.pdf]

**Supplementary information**

**Table of Contents**


**Supplementary Table 1.** Antimicrobials received by No LRTI group patients. *Page 2*

**Appendix 1**. Final GPT-4 prompt for diagnosis of LRTI *Page 3*

**Appendix 2**. Example of GPT-4 output for diagnosis of LRTI *Pages 4-6*

**Appendix 3**. Example of GPT-4 output for diagnosis of No LRTI *Pages 7-9*

**Appendix 4**. CXR reads of patients diagnosed with LRTI by GPT-4 but not by comparison physicians. *Pages 10-11*

**Supplementary Table 1. Antimicrobials received by No LRTI group patients.**

| | **Derivation Cohort** | **Validation Cohort** |
|---|---|---|
| | N=49 | N=22 |
| **Class of LRTI-directed antimicrobials received** (No., %) | | |
| Antibacterial agent(s) | 20 (41%) | 7 (32%) |
| Antiviral agent(s) | 0 (0%) | 0 (0%) |
| Antifungal agent(s) | 0 (0%) | 0 (0%) |
| Multiple pathogen classes treated | 4 (8%) | 0 (0%) |
| Not treated for LRTI | 25 (51%) | 15 (68%) |

Antimicrobials were not considered treatment for LRTI if administered for established non-pulmonary infections or for prophylaxis. Four No LRTI patients in the derivation cohort received antimicrobials directed at more than one class of pathogen; three of whom received joint antifungal and antibacterial treatment, and one of whom received antiviral and antibacterial treatment.

**Appendix 1. Final GPT-4 prompt for diagnosis of LRTI.**

You are an AI clinician with expertise in critical care and/or infectious diseases. You are trying to adjudicate whether a patient has lower respiratory tract infection (LRTI).
Below, you will be given the most recent chest X-ray result (CXR) of a patient, and the clinician note for that patient prior to the most recent CXR. The note might contain information on the previous CXRs as well.
I want you to come to your own independent adjudication, regardless of what the clinical team thought. Therefore, in your adjudication, you should ignore the antibiotics treatment the patient was receiving in your analysis. You should also ignore the clinical team's LRTI diagnosis in your analysis.
I want you to give an answer in two sections. In the first section, called "Analysis", you analyze the provided information step by step to determine if the patient has LRTI, and present your analysis in a numbered list format. In the second section, called "Adjudication", you weigh the evidence for and against LRTI, then decide the patient is more likely to have LRTI or not by choosing one of two options, "LRTI" or "no LRTI", to be your final conclusion.

Here is the CXR:
*[copy paste the CXR from the docx file here, remove any trailing new lines at the end of the text]*

Here is the note:
*[copy paste the note from the docx file here, remove any trailing new lines at the end of the text]*

**Appendix 2. Example of GPT-4 output for diagnosis of LRTI.**

**Patient 409**

| **Versa Chat settings:**<br>Engine: Azure OpenAI<br>Data Source: OpenAI Web<br>Model: GPT-4 Turbo<br>Temperature: 0.2<br>Conversation mode: enabled |
|---|

Attempt 1

Analysis:

1. **Chest X-ray (CXR) Findings:**
    - The most recent CXR indicates the presence of a left lower lobe collapse.
    - There is also a note of worsening right lower lobe consolidation, which could be due to aspiration or pneumonia.
2. **Clinical History:**
    - The patient has been admitted to the hospital for an extended period and has had multiple interventions, including intubation and extubation, valve adjustments, and surgeries related to neurocysticercosis and ventriculoperitoneal shunt (VPS) complications.
    - The patient experienced a PEA arrest, which could have led to periods of hypoxia, potentially predisposing them to infections.
    - The patient has had a recent surgical intervention (hematoma evacuation and revision of VPS) which could be a source of infection or could complicate the clinical picture.
3. **Vital Signs:**
    - The patient's temperature has remained within normal limits, which may not support the presence of an active infection.
    - The heart rate is elevated (126 bpm), which could be a sign of systemic stress, possibly due to infection.
    - Respiratory rate is within normal limits.
    - Blood pressure is slightly elevated.
    - Oxygen saturation is at 95%, which is at the lower end of the normal range and could indicate some respiratory compromise.
4. **Physical Exam:**
    - The patient is nonverbal, which limits the ability to obtain symptoms directly related to respiratory distress or infection.
    - Neurological status seems to be the focus of the patient's assessment and care, given the history of neurocysticercosis and recent neurosurgical interventions.
5. **Laboratory and Other Clinical Information:**
    - There is no mention of white blood cell count or other laboratory markers that could indicate infection.
    - The patient is on dexamethasone, which could mask signs of infection due to its anti-inflammatory effects.
    - There is no mention of sputum culture or other microbiological tests that could confirm the presence of an LRTI.
6. **Other Considerations:**
    - The patient's history of neurocysticercosis and recent neurosurgical interventions could lead to an immunocompromised state, increasing the risk for infections, including LRTI.

- The presence of an endotracheal tube and recent surgeries could increase the risk of nosocomial infections.

Adjudication:
Based on the analysis above, the evidence suggesting the presence of an LRTI includes the worsening right lower lobe consolidation noted on the CXR, which could represent aspiration or pneumonia, and the elevated heart rate which might indicate systemic stress from an infection. However, the lack of fever, normal respiratory rate, and the absence of leukocytosis or purulent sputum in the information provided make the diagnosis of LRTI less certain.
Considering the balance of the evidence, the patient is more likely to have an LRTI, primarily due to the radiological findings of consolidation and collapse, which are consistent with this diagnosis, despite the absence of some clinical signs typically associated with infection.
Therefore, the final conclusion is:
**LRTI**

<u>Attempt 2</u>
Analysis

1. **Chest X-ray (CXR) Findings:**
    - The CXR from **[date redacted]** shows an endotracheal tube in place, terminating 34 mm above the carina, which is appropriate placement.
    - There is an interval development of left lower lobe collapse since the comparison CXR on the same day.
    - There is worsening right lower lobe consolidation, which could be due to aspiration or pneumonia.
    - The cardiomediastinal silhouette remains unchanged.
2. **Clinical History:**
    - The patient was admitted on **[date redacted]** and has had a complicated hospital course including intubation, extubation, ventriculoperitoneal shunt (VPS) replacement, a PEA arrest with successful resuscitation, and multiple surgical interventions for neurocysticercosis and its complications.
    - The patient has had a recent surgery for hematoma evacuation and revision of the VPS on **[date redacted]**
3. **Vital Signs:**
    - The patient’s temperature has been within normal limits.
    - The heart rate is elevated at 126 bpm.
    - Respiratory rate is within normal limits at 18 breaths per minute.
    - Blood pressure is at the higher end of the recorded range but not hypertensive.
    - Oxygen saturation is at 95%, which is within normal limits but on the lower end of the expected range for someone on supplemental oxygen.
4. **Physical Exam:**
    - The patient is nonverbal, which could be due to underlying neurological conditions or sedation.
    - Pupils are sluggishly reactive, which could indicate central nervous system involvement.
    - The patient is following commands with the right upper extremity and moving both lower extremity toes spontaneously.
5. **Problem-based Assessment & Plan:**
    - The patient has a history of neurocysticercosis with recent neurosurgical interventions.
    - There is mention of cerebral edema being treated with dexamethasone.
    - The head of the bed is maintained at >45 degrees, likely for both neuroprotective reasons and aspiration prevention.

- Tube feeds have been restarted, indicating the patient is receiving enteral nutrition.

6. **Other Considerations:**
   - The patient has had a recent PEA arrest secondary to a pulmonary embolism, which could predispose to aspiration events.
   - The patient is on dexamethasone, which can suppress immune responses and increase the risk of infection.
   - The patient's recent tachycardia and hypertension were relieved by a bowel movement, suggesting these symptoms were not due to infection.

Adjudication

Based on the analysis of the provided CXR and clinical notes, the evidence for LRTI includes the worsening right lower lobe consolidation and the new left lower lobe collapse. The patient's elevated heart rate could also support the presence of an infection. However, the normal temperature and the fact that the patient's tachycardia was relieved by a non-infectious intervention suggest that the heart rate may not be due to an infection. The unchanged cardiomediastinal silhouette does not provide additional support for LRTI.

Given the evidence, the patient is more likely to have **LRTI** due to the radiographic findings of worsening consolidation and new collapse, which are consistent with infectious processes in the lung, especially in the context of recent aspiration risks and immunosuppression from steroids.

<u>Attempt 3</u>

Analysis

1. **Chest X-ray (CXR) Findings:**
   - The CXR indicates that there is a new development of left lower lobe collapse.
   - There is also a noted worsening of right lower lobe consolidation, which could be due to aspiration or pneumonia.
2. **Clinical History:**
   - The patient has a complicated medical history with neurocysticercosis, multiple shunt failures, and a pulmonary embolism (PE) arrest.
   - The patient has undergone multiple surgical interventions, including ventriculoperitoneal shunt (VPS) revisions and hematoma evacuation.
   - The patient has been intubated and extubated multiple times, which increases the risk for aspiration and subsequent pneumonia.
3. **Vital Signs:**
   - The patient's temperature is within normal limits, which may or may not rule out infection.
   - Tachycardia is present (heart rate of 126), which can be a sign of infection but can also be due to other causes.
   - The respiratory rate is within normal limits.
   - Blood pressure is slightly elevated, which could be due to various reasons, including stress or pain.
   - Oxygen saturation is at 95%, which is at the lower end of the normal range and could suggest some degree of respiratory compromise.
4. **Physical Exam:**
   - The patient is nonverbal, which limits the ability to obtain symptoms such as cough or dyspnea.
   - Neurological status is compromised due to underlying neurocysticercosis and recent surgeries, which may mask typical signs and symptoms of LRTI.
5. **Recent Interventions and Treatments:**

- The patient has had a recent external ventricular drain (EVD) placement and revisions of the VPS, which can be associated with infection risk, but not specifically LRTI.
   - The patient is on dexamethasone, which can suppress the immune response and potentially mask or exacerbate an infection.
   - The patient has had recent mobilization and physical therapy, which can help prevent respiratory infections by improving lung expansion and secretion clearance.
6. **Laboratory and Imaging:**
   - No recent blood work is provided to assess for markers of infection such as white blood cell count or inflammatory markers.
   - The most recent head CT and MRI do not provide information about the lower respiratory tract.
7. **Risk Factors for LRTI:**
   - The patient has several risk factors for LRTI, including recent intubation, neurosurgical procedures, and a history of PE arrest, which could predispose to aspiration.

Adjudication

Given the evidence presented:

- The new development of left lower lobe collapse and worsening right lower lobe consolidation on CXR are radiographic findings that can be associated with LRTI.
- The patient's history of intubation and neurosurgical procedures increases the risk for aspiration pneumonia.
- The absence of fever does not rule out LRTI, especially in the context of immunosuppression due to steroids.
- Tachycardia could be a nonspecific sign of infection.

**Conclusion: LRTI**

The radiographic findings, combined with the patient's clinical history and risk factors, support the diagnosis of a lower respiratory tract infection more than any alternative diagnosis provided in the information.

**Appendix 3. Example of GPT-4 output for diagnosis of No LRTI.**

**Patient 1325**

**Versa Chat settings:**
Engine: Azure OpenAI
Data Source: OpenAI Web
Model: GPT-4 Turbo
Temperature: 0.2
Conversation mode: enabled

Attempt 1

Analysis

1. **Chest X-ray Findings**: The most recent CXR dated **[date redacted]** shows an increased size of a small to moderate right pleural effusion and an unchanged size of a large left pleural effusion, along with interstitial edema. No pneumothorax is noted.
2. **Comparison with Previous CXRs**:
   - The CXR from **[date redacted]** reported unchanged pleural effusions and interstitial edema compared to the previous day.
   - The CXR from **[date redacted]** noted stable pleural effusions and probable moderate interstitial pulmonary edema.
3. **Clinical Context and History**:
   - The patient has a history of heart failure (HFrEF), ventricular septal defect (VSD), pulmonary hypertension, and recent diagnosis of atrial fibrillation.
   - Recent hospital events include episodes of ventricular tachycardia, hypotension, and bradycardia, managed with various medications including amiodarone and vasopressors.
4. **Symptoms and Physical Examination**:
   - The patient is intubated and sedated, limiting symptom assessment.
   - Physical examination notes decreased breath sounds anteriorly, which could be due to pleural effusions.
5. **Laboratory and Microbiology Results**:
   - Elevated white blood cell count (WBC) around 13.5, which could indicate infection but is not specific.
   - Urine culture positive for Klebsiella pneumoniae, indicating a urinary tract infection.
   - Peripheral blood cultures showed no growth, reducing the likelihood of bacteremia.
6. **Other Relevant Findings**:
   - The patient is on multiple medications that could influence fluid balance and cardiac function.
   - The patient has been managed for fluid overload, which is a likely contributor to the pleural effusions.

Adjudication

Given the clinical context of heart failure with documented fluid overload, the presence of bilateral pleural effusions and interstitial edema is more likely related to the patient’s cardiac status rather than an infectious process. The lack of significant changes in the CXR over a short period, absence of new clinical signs specific to pneumonia (e.g., fever, leukocytosis specific to infection, productive cough), and the management focused on cardiac and fluid issues support this interpretation. The positive urine culture indicates a urinary source of infection rather than respiratory.

**Conclusion: no LRTI**. The findings are more consistent with complications of heart failure and fluid overload rather than a lower respiratory tract infection.

Attempt 2

Analysis

1. **Clinical Context and History**: The patient is a 64-year-old female with a history of heart failure with reduced ejection fraction (HFrEF), ventricular septal defect (VSD) due to myocardial infarction, pulmonary hypertension, and recent diagnosis of atrial fibrillation. She presents with symptoms of shock and has been managed with multiple interventions including intubation, vasopressors, and antiarrhythmic drugs.
2. **Chest X-ray Findings**: The most recent chest X-ray (CXR) dated **[date redacted]** shows:
    - Increased size of a small to moderate right pleural effusion.
    - Unchanged size of a large left pleural effusion.
    - Interstitial edema.
    - Presence of an endotracheal tube, enteric tube, right internal jugular venous catheter, and epicardial pacing leads.
3. **Comparison with Previous CXRs**: Comparing the CXR from **[date redacted]** and [**date redacted]**:
    - There is a progression in the size of the right pleural effusion.
    - The left pleural effusion and interstitial edema have remained unchanged.
    - No evidence of pneumothorax in any of the CXRs.
4. **Clinical Notes and Observations**:
    - The patient has been managed for bradycardia, hypotension, and hyperkalemia, with concerns for digoxin toxicity.
    - She has experienced intermittent runs of ventricular tachycardia.
    - She is intubated and sedated, with decreased breath sounds anteriorly and significant bilateral lower extremity edema.
5. **Laboratory Results**:
    - Elevated white blood cell count (WBC) at 13.5, which could indicate an infection.
    - Elevated creatinine levels suggesting acute kidney injury, possibly related to cardiorenal syndrome or fluid overload.
    - Urine culture positive for Klebsiella pneumoniae, indicating a urinary tract infection.
6. **Microbiology and Infectious Concerns**:
    - The presence of Klebsiella pneumoniae in the urine culture indicates an active infection, though this does not necessarily confirm a respiratory infection.
    - Blood cultures showed no growth, reducing the likelihood of a systemic bacterial infection.
7. **Symptoms and Signs Suggestive of LRTI**:
    - The increase in pleural effusion size, particularly on the right side, could suggest an infectious process, but it is also consistent with fluid overload in the context of heart failure.
    - The patient’s clinical presentation includes elements that could be explained by both cardiac and infectious etiologies.

Adjudication

Considering the evidence:

- The progression of pleural effusion could be secondary to heart failure exacerbated by fluid overload rather than a direct infectious cause.

- The lack of systemic infection signs in blood cultures and the primary findings of congestive symptoms and fluid management issues lean towards a non-infectious etiology of the respiratory findings.

**Conclusion: no LRTI**

The findings are more consistent with complications related to heart failure and fluid overload rather than a lower respiratory tract infection.

<u>Attempt 3</u>

Analysis

1. **Clinical Context and History**: The patient is a 64-year-old female with a history of heart failure with reduced ejection fraction (HFrEF), ventricular septal defect (VSD) secondary to myocardial infarction, pulmonary hypertension, and newly diagnosed atrial fibrillation. She was admitted with symptoms of digoxin toxicity, bradycardia, and hypotension, and has experienced episodes of ventricular tachycardia requiring resuscitation.
2. **Chest X-ray Findings**: The most recent chest X-ray (CXR) dated **[date redacted]** shows an increased size of a small to moderate right pleural effusion, unchanged size of a large left pleural effusion, and interstitial edema. There is no evidence of pneumothorax.
3. **Comparison with Previous CXRs**: The CXR from [**date redacted]** and [**date redacted]** also documented large left and small right pleural effusions with interstitial edema, indicating a persistent pattern of fluid accumulation in the pleural spaces and lung interstitium.
4. **Symptoms and Physical Examination**: The patient is intubated and sedated, with decreased breath sounds anteriorly, significant bilateral lower extremity edema, and a history of acute hypercarbic respiratory failure. These findings suggest significant fluid overload and compromised pulmonary function.
5. **Laboratory Results**: Elevated white blood cell count (WBC) around 13.5, which could indicate an inflammatory or infectious process. The patient also has elevated creatinine levels suggesting renal impairment, which can contribute to fluid overload.
6. **Microbiology Results**: A urine culture grew 100,000 colonies of Klebsiella pneumoniae, indicating a urinary tract infection. Blood cultures showed no growth, reducing the likelihood of bacteremia.
7. **Cardiovascular and Respiratory Management**: The patient is on multiple medications including diuretics (bumetanide), which are being used to manage fluid overload. The presence of pleural effusions and interstitial edema is likely exacerbated by her cardiac conditions and fluid management challenges.
8. **Infectious Disease Consideration**: Despite the presence of a urinary tract infection, there is no direct evidence from the clinical notes or CXR that suggests a lower respiratory tract infection (LRTI). The pleural effusions and interstitial edema are more likely related to her cardiac status and fluid management.

Adjudication

Based on the analysis of the CXR, clinical notes, and laboratory results, the findings are more consistent with complications related to heart failure exacerbation and fluid overload rather than a primary infectious process in the lower respiratory tract. The increased pleural effusions and interstitial edema are typical in patients with acute or exacerbated heart failure and do not necessarily indicate an LRTI.

**Conclusion: no LRTI**

**Appendix 4. CXR radiologist reads of patients diagnosed with LRTI by GPT-4 but not by comparison physicians.** Underlines added.

**Patient 270**
Comparison Physician LRTI score: 0/3
Versa LRTI Score: 2/3
CXR Radiology Impression: The endotracheal tube terminates 3.9 cm from the carina. Right dual-lead pacemaker is unchanged. Increased patchy multifocal consolidation predominantly involving the right upper and right lower lobes which may represent asymmetric pulmonary edema or multifocal pneumonia. No change in cardiomediastinal silhouette.
Clinical note suggests LRTI as a possible diagnosis: No

**Patient 278**
Comparison Physician LRTI score: 1/3
Versa LRTI Score: 3/3
CXR Radiology Impression: Endotracheal tube tip approximately 2.4 cm above the carina. Significantly improved aeration of the left apex. The left base remains completely opacified. Hazy opacity in the right base is slightly worse, likely worsened atelectasis.
Clinical note suggests LRTI as a possible diagnosis: Yes

**Patient 279**
Comparison Physician LRTI score: 1/3
Versa LRTI Score: 3/3
CXR Radiology Impression: Right chest port is unchanged in position. Worsening left basilar opacity, which could be due to atelectasis, aspiration or pneumonia.
Clinical note suggests LRTI as a possible diagnosis: No

**Patient 314**
Comparison Physician LRTI score: 1/3
Versa LRTI Score: 3/3
CXR Radiology Impression: Interval increase in right pleural effusion. Interval worsening bilateral central peribronchial vascular opacities, with prominent pulmonary artery, compatible with worsening pulmonary edema. Stable cardiomegaly. Stable tubes and lines. No definite fracture.
Clinical note suggests LRTI as a possible diagnosis: Yes

**Patient 360**
Comparison Physician LRTI score: 0/3
Versa LRTI Score: 3/3
CXR Radiology Impression: Endotracheal tube which is partially obscured by surgical hardware with the tip approximately 4.5 cm above the carina. Posterior spinal fusion hardware involving the lower cervical and thoracic spine with associated surgical drain. No pneumothorax. Left lower lobe collapse. Ill-defined opacities throughout the left lung likely represents aspiration, asymmetric pulmonary edema, or infection. Minimal right basilar atelectasis. Unremarkable cardiomediastinal silhouette.
Clinical note suggests LRTI as a possible diagnosis: No

**Patient 386**
Comparison Physician LRTI score: 1/3
Versa LRTI Score: 3/3

CXR Radiology Impression: Endotracheal tube, enteric tube, and right PICC remain in place. Low lung volumes with slight worsening of retrocardiac opacity which may reflect atelectasis, pneumonia, or aspiration. Small left pleural effusion has increased in size. Slightly improved right basilar atelectasis. Unchanged cardiomediastinal silhouette.
Clinical note suggests LRTI as a possible diagnosis: No

**Patient 409**
Comparison Physician LRTI score: 0/3
Versa LRTI Score: 3/3
CXR Radiology Impression: Endotracheal tube has been placed, terminating 34 mm above the carina. Interval development of left lower lobe collapse. Worsening right lower lobe consolidation, could represent aspiration or pneumonia. Unchanged cardiomediastinal silhouette
Clinical note suggests LRTI as a possible diagnosis: No

**Patient 513**
Comparison Physician LRTI score: 1/3
Versa LRTI Score: 3/3
CXR Radiology Impression: Tip of endotracheal tube is 2 cm above the carina. Decreased lung volumes. Improvement in interstitial pulmonary edema.
Clinical note suggests LRTI as a possible diagnosis: No

**Patient 579**
Comparison Physician LRTI score: 1/3
Versa LRTI Score: 3/3
CXR Radiology Impression: Endotracheal tube has been placed, with tip at approximately 4.5 cm above the carina. Right central line remains in place. Interval development of left lower lobe opacities which could represent aspiration or pneumonia. A small left-sided pleural effusion has also developed. Unchanged cardiomediastinal silhouette
Clinical note suggests LRTI as a possible diagnosis: No
