# Supplementary material for: Integrating a host biomarker with a large language model for diagnosis of lower respiratory tract infection
Source: Nat Commun. 2025 Dec 16;16:10882. doi: 10.1038/s41467-025-66218-5 (PMC12708615; doi:10.1038/s41467-025-66218-5)
Supplement: Supplementary file 3 — Reporting Summary [file 41467_2025_66218_MOESM3_ESM.pdf]

nature portfolio

Corresponding author(s): Natasha Spottiswoode

Last updated by author(s): Oct 19, 2025

Reporting Summary

Nature Portfolio wishes to improve the reproducibility of the work that we publish. This form provides structure for consistency and transparency in reporting. For further information on Nature Portfolio policies, see our Editorial Policies and the Editorial Policy Checklist.

Statistics

For all statistical analyses, confirm that the following items are present in the figure legend, table legend, main text, or Methods section.

n/a	Confirmed	
☐	☒	The exact sample size (*n*) for each experimental group/condition, given as a discrete number and unit of measurement
☐	☒	A statement on whether measurements were taken from distinct samples or whether the same sample was measured repeatedly
☐	☒	The statistical test(s) used AND whether they are one- or two-sided<br>*Only common tests should be described solely by name; describe more complex techniques in the Methods section.*
☒	☐	A description of all covariates tested
☒	☐	A description of any assumptions or corrections, such as tests of normality and adjustment for multiple comparisons
☐	☒	A full description of the statistical parameters including central tendency (e.g. means) or other basic estimates (e.g. regression coefficient) AND variation (e.g. standard deviation) or associated estimates of uncertainty (e.g. confidence intervals)
☐	☒	For null hypothesis testing, the test statistic (e.g. *F*, *t*, *r*) with confidence intervals, effect sizes, degrees of freedom and *P* value noted<br>*Give P values as exact values whenever suitable.*
☒	☐	For Bayesian analysis, information on the choice of priors and Markov chain Monte Carlo settings
☒	☐	For hierarchical and complex designs, identification of the appropriate level for tests and full reporting of outcomes
☒	☐	Estimates of effect sizes (e.g. Cohen's *d*, Pearson's *r*), indicating how they were calculated

Our web collection on statistics for biologists contains articles on many of the points above.

Software and code

Policy information about availability of computer code

Data collection	Study data was collected using REDCap LTS Version 15.0.29
Data analysis	Statistical and classifier performance analyses were carried out in R version 4.5.0. Electronic medical records were analyzed using Open AI GPT-4 as implemented in Versa, which is a University of California San Francisco Health Insurance Portability and Accountability Act (HIPAA) compliant large language model. All data and code are available at https://github.com/infectiousdisease-langelier-lab/LRTI_FABP4_GPT4_classifier.

For manuscripts utilizing custom algorithms or software that are central to the research but not yet described in published literature, software must be made available to editors and reviewers. We strongly encourage code deposition in a community repository (e.g. GitHub). See the Nature Portfolio guidelines for submitting code & software for further information.

Data

Policy information about availability of data

All manuscripts must include a data availability statement. This statement should provide the following information, where applicable:

- Accession codes, unique identifiers, or web links for publicly available datasets
- A description of any restrictions on data availability
- For clinical datasets or third party data, please ensure that the statement adheres to our policy

All data and code used in this study are available at: https://github.com/infectiousdisease-langelier-lab/LRTI_FABP4_GPT4_classifier.

Research involving human participants, their data, or biological material

Policy information about studies with human participants or human data. See also policy information about sex, gender (identity/presentation), and sexual orientation and race, ethnicity and racism.

Reporting on sex and gender	We provide in Table 1 a de-identified summary of the sex assigned at birth of patients in our derivation and validation cohorts. Sex assigned at birth is compared between patients with and without lower respiratory tract infection, as with other demographics in our cohort.
Reporting on race, ethnicity, or other socially relevant groupings	We provide in Table 1 a de-identified summary of the self-identified race of patients in our derivation and validation cohorts. Self-identified race is compared between patients with and without lower respiratory tract infection, as with other demographics in our cohort.
Population characteristics	This observational study evaluated adults with acute respiratory failure requiring mechanical ventilation who were admitted to the University of California, San Francisco intensive care units.
Recruitment	Subjects were enrolled sequentially between 10/2013-03/2020 (derivation cohort) and 04/2020-12/2023 (validation cohort), within the first 72 hours of intubation for respiratory failure. The UCSF Institutional Review Board (IRB) approved an initial waiver consent for obtaining excess respiratory fluid, blood, and urine samples, and informed consent was subsequently obtained from patients or their surrogates for continued study participation according to IRB protocol #10-02701 for the derivation cohort, and #20-30497 and #10-02852 for validation cohort. For patients whose surrogates provided informed consent, follow-up consent was then obtained if patients survived their acute illness and regained the ability to consent. For subjects who died before consent could be obtained from either patient or surrogate, a full waiver of consent was approved. For all surviving subjects, if consent was not eventually obtained from either patient or surrogate, all specimens and data were destroyed.
Ethics oversight	This research was approved by the University of California, San Francisco Institutional Review Board (IRB) under the following protocols: #10-02701 for the derivation cohort, and #20-30497 and #10-02852 for validation cohort.<br><br>If a patient met inclusion criteria for these studies, then a study coordinator or physician obtained written informed consent for enrollment from the patient or their surrogate. Patients or their surrogates were provided with detailed written and verbal information about the goals of the study, the data and specimens that would be collected, and the potential risks to the subject. Patients and their surrogates were also informed that there would be no benefit to them from being enrolled in these studies and that they may withdraw informed consent at any time during the course of the study. All questions were answered, and informed consent documented by obtaining the signature of the patient or their surrogate on the consent document (or during the COVID-19 pandemic, the IRB-approved electronic equivalent, to enable touchless consent).<br><br>Many critically ill patients are unconscious at the time of intensive care unit (ICU) admission due to their underlying illness and/or are endotracheally intubated for airway management or acute respiratory failure. The patients who are not unconscious are often in pain and may have acute delirium due to critical illness and/or medications. For these reasons, many subjects are unable to provide informed consent at the time of enrollment. Because these studies could not practically be done otherwise and was deemed to be minimal risk by the UCSF IRB, if a patient was unable and a surrogate was not available to provide consent, patients were enrolled with waiver of initial consent.<br><br>Specifically, for subjects who were unable to provide informed consent at the time of enrollment, our study team was permitted to collect biological samples as well as clinical data from the medical record obtained prior to consent. Surrogate consent was vigorously pursued for all patients; moreover, each patient was regularly examined to determine if and when s/he was able to consent for him/herself, and the nursing and ICU staff were contacted daily for information about surrogates' availability. For patients whose surrogates provided informed consent, follow-up consent was subsequently obtained from the patient if they survived their acute illness and regained the ability to consent. For subjects who died prior to the consent being obtained, a full waiver of consent was approved by the UCSF IRB for both cohort studies. Lack of a surrogate to provide consent is common in critically ill patients. To address this, the UCSF IRB also approved a full waiver of consent for subjects in these cohorts who remained unable to provide informed consent and had no contactable surrogate identified within 28 days. Before utilizing this waiver, we made and documented at least three separate attempts to identify and contact the patient or surrogate over a month-long period. For all surviving subjects, if consent was not eventually obtained from either patient or surrogate, all specimens and data were destroyed.<br><br>No personally identifiable information has been included as part of this manuscript for any enrolled patients.

Note that full information on the approval of the study protocol must also be provided in the manuscript.

Field-specific reporting

Please select the one below that is the best fit for your research. If you are not sure, read the appropriate sections before making your selection.

- [x] Life sciences
- [ ] Behavioural & social sciences
- [ ] Ecological, evolutionary & environmental sciences

For a reference copy of the document with all sections, see nature.com/documents/nr-reporting-summary-flat.pdf

Life sciences study design

All studies must disclose on these points even when the disclosure is negative.

Sample size	Sample sizes were based on the number of patients recruited in each cohort who met gold-standard multi-physician adjudication as having lower respiratory tract infection or a non infectious cause of respiratory failure.
Data exclusions	As detailed in Figure 1, patients were excluded if they had no clinical note available for analysis. Five patients whose clinical notes were used for prompt engineering were also excluded.
Replication	We replicated our results by performing analyses in an independent validation cohort.
Randomization	This was an observational study, patients were not randomized to any interventions.
Blinding	All physician reviewers ascertaining the lower respiratory tract infection status (infection present, or infection absent) were blinded to diagnostic classifier results including GPT-4 model outputs.

Reporting for specific materials, systems and methods

We require information from authors about some types of materials, experimental systems and methods used in many studies. Here, indicate whether each material, system or method listed is relevant to your study. If you are not sure if a list item applies to your research, read the appropriate section before selecting a response.

Materials & experimental systems

n/a	Involved in the study	
☒	☐	Antibodies
☒	☐	Eukaryotic cell lines
☒	☐	Palaeontology and archaeology
☒	☐	Animals and other organisms
☐	☒	Clinical data
☒	☐	Dual use research of concern
☒	☐	Plants

Methods

n/a	Involved in the study	
☒	☐	ChIP-seq
☒	☐	Flow cytometry
☒	☐	MRI-based neuroimaging

Clinical data

Policy information about clinical studies

All manuscripts should comply with the ICMJE guidelines for publication of clinical research and a completed CONSORT checklist must be included with all submissions.

Clinical trial registration	N/A; observational study.
Study protocol	UCSF Institutional Review Board protocols #10-02701, #20-30497 and #10-02852.
Data collection	Patients were recruited from a tertiary hospital intensive care unit between 2013 and 2023.
Outcomes	Our pre-defined outcome was accurate diagnosis of lower respiratory tract infection based on a gold-standard of multi-physician adjudication.

Plants

Seed stocks	*Report on the source of all seed stocks or other plant material used. If applicable, state the seed stock centre and catalogue number. If plant specimens were collected from the field, describe the collection location, date and sampling procedures.*
Novel plant genotypes	*Describe the methods by which all novel plant genotypes were produced. This includes those generated by transgenic approaches, gene editing, chemical/radiation-based mutagenesis and hybridization. For transgenic lines, describe the transformation method, the number of independent lines analyzed and the generation upon which experiments were performed. For gene-edited lines, describe the editor used, the endogenous sequence targeted for editing, the targeting guide RNA sequence (if applicable) and how the editor was applied.*
Authentication	*Describe any authentication procedures for each seed stock used or novel genotype generated. Describe any experiments used to assess the effect of a mutation and, where applicable, how potential secondary effects (e.g. second site T-DNA insertions, mosiacism, off-target gene editing) were examined.*
